# Non-invasive localization of atrial ectopic beats by using simulated body surface P-wave integral maps

**DOI:** 10.1371/journal.pone.0181263

**Published:** 2017-07-13

**Authors:** Ana Ferrer-Albero, Eduardo J. Godoy, Miguel Lozano, Laura Martínez-Mateu, Felipe Atienza, Javier Saiz, Rafael Sebastian

**Affiliations:** 1 Centro de Investigación e Innovación en Bioingeniería (Ci2B), Universitat Politècnica de València, Valencia, Spain; 2 Computational Multiscale Physiology Lab (CoMMLab), Department of Computer Science, Universitat de Valencia, Valencia, Spain; 3 Hospital Gregorio Marañón, Madrid, Spain; Universita degli Studi di Roma La Sapienza, ITALY

## Abstract

Non-invasive localization of continuous atrial ectopic beats remains a cornerstone for the treatment of atrial arrhythmias. The lack of accurate tools to guide electrophysiologists leads to an increase in the recurrence rate of ablation procedures. Existing approaches are based on the analysis of the P-waves main characteristics and the forward body surface potential maps (BSPMs) or on the inverse estimation of the electric activity of the heart from those BSPMs. These methods have not provided an efficient and systematic tool to localize ectopic triggers. In this work, we propose the use of machine learning techniques to spatially cluster and classify ectopic atrial foci into clearly differentiated atrial regions by using the body surface P-wave integral map (BSPiM) as a biomarker. Our simulated results show that ectopic foci with similar BSPiM naturally cluster into differentiated non-intersected atrial regions and that new patterns could be correctly classified with an accuracy of 97% when considering 2 clusters and 96% for 4 clusters. Our results also suggest that an increase in the number of clusters is feasible at the cost of decreasing accuracy.

## Introduction

The non-invasive localization of atrial ectopic foci during focal atrial tachycardia is complex to determine. Methods based on either standard 12-lead electrocardiograms (ECG) or body surface potential maps (BSPM) have not shed sufficient light on how to accurately correlate ectopic focus locations with the distribution of potential and P-wave characteristics on the torso surface. This limitation results in longer intra-operative invasive mapping procedures, which could lead to suboptimal localization and ablation of focal triggers. The last consequence is an increased recurrence rate of ablation procedures to stop the arrhythmia.

From the early 70’s, several approaches have been proposed to localize ectopic foci in the atria. The first studies in humans consisted in pacing the atria at different sites to analyse the morphology, time sequence and polarity of P-waves [1–3] using the standard 12-lead ECG. Unfortunately, the usability of standard 12-lead ECG for such analysis is poor due to the number of P-waves with low amplitude or isoelectric morphology. In the late 90’s, some studies increased the number of body surface electrodes and focused on studying the body surface potential distribution [4–7] or the body surface integral P-wave maps (BSPiMs) [8–11] during controlled atrial pacing. Those approaches provided dense maps of P-wave signals, triggered from stimulation catheters placed at a few locations inside the atria that allowed characterising the signals and analysing their distribution on the torso surface. More recently, a combination of BSPM with surface wavefront propagation maps [12], and with the spatial evolution of the electrical dipole [13] have been proposed to get an insight into the determination of the ectopic focus origin that maintains the arrhythmia and the dynamics of the dipole of atrial depolarization prior to the ablation procedure. However, none of those approaches based on the forward problem have provided an efficient and systematic method to localize the specific origin of the ectopic excitation.

Recent studies have focused on designing and validating algorithms based on P-wave polarity that used 12-lead [14,15] or 64-lead recordings [16] for identifying the origin of ectopic sites that were artificially induced from a pacing catheter in the atria. Those procedures showed a general good accuracy for detecting the site of some ectopic foci. However, their accuracy decreases again when bi-phasic P-waves are present in many leads [16]. Computational tools have also been proposed to help in the localization of ectopic foci. For instance, an approach known as electrocardiographic imaging (ECGi) allows estimating the electric activity of the heart by solving the inverse problem, i.e., it reconstructs maps of epicardial potentials from data measured on the body surface. This method has evolved from the first non-human study in the late 90’s where a dog heart was paced at different sites on the ventricles [17] for the diagnostics of focal ventricular tachycardia [18], the characterization of epicardial atrial pathological activation [19] or the guidance of atrial fibrillation ablation [20]. Further methods propose to build a database of simulated BSPM combined with machine learning techniques to predict the localization of ventricular ectopic triggers [21], or the use of machine learning techniques to classify cardiac excitation patterns during atrial fibrillation using a cross-validated support vector machine (SVM) [22]. Taking advantage of the methodologies and results provided by these two last studies, in this work, we use our detailed anatomical and electrophysiological multi-scale atria-torso model [23–25] together with similar machine learning techniques (Kernel regressions and SVM) to develop a pipeline to cluster and classify BSPiMs into groups associated with ectopic atrial sites. The goal is, first, to spatially cluster ectopic atrial foci into clearly differentiated atrial regions based on the analysis of the BSPiMs that they display; second, to classify new BSPiMs into the previously well-defined clusters (related to atrial locations); finally, to provide a virtual population of 58 in-silico normalized reference BSPiM patterns to gain insight into the different patterns computed by stimulating across both atria.

## Material and methods

### Multiscale atrial-torso model

We made use of our three-dimensional model of the human atria described in [23], which was developed by using anatomical and histological data gathered over several years from 30 formalin-fixed healthy hearts [26–29]. This atrial finite element model consists of a multi-layer mesh with a wall thickness in the range of ~600–900 μm. It includes heterogeneous fibre direction in 21 atrial regions, electrophysiological heterogeneity in 8 different regions modelled by adjusting I_to_, I_CaL_ and I_Kr_ in Maleckar’s model [30], and tissue heterogeneity modelled by specific conduction velocities and anisotropy ratios (see [23] for further details). Regarding the torso model, we also used our previous version [23] based on data in the open access and anonymized repository made available online by the Centre for Integrative Biomedical Computing (CBIC) from University of Utah [31]. This model consists of the main organs (lungs, bones, liver, ventricle, blood pools, and flesh) with their specific conduction properties and was segmented by using the Seg3D software [32] and meshed with TetGen [33]. Both models are available at RIUNET open repository (http://hdl.handle.net/10251/55150).

In this work, we have enhanced this previous detailed multiscale atria-torso model [23] to incorporate more realistic electrophysiology properties at cellular and tissue level [24,25] and to improve the interpolation between scales. The anatomical atrial domain consists of a 3D mesh with 754.893 nodes and 515.005 linear hexahedral elements, and a regular spatial resolution of 300 μm. Similar to previous studies [23,34–36], we used a thin and homogeneous wall thickness in order to reduce the mesh size and computation load. This property is expected to slightly influence the activation maps. However, since we look for the relationship between BSPiMs and the atrial ectopic sites, it seems likely that increasing the atrial wall thickness homogeneously will not affect such association.

The Courtemanche-Ramírez-Nattel (CRN) ionic model for human atrial electrophysiology has been used to simulate the action potentials [37]. From its original formulation, 9 different cellular sub-models have been derived to account on the heterogeneous action potential (AP) morphologies in the atria under normal physiological conditions. In this regard, we have split the Bachmann’s bundle (BB) in its right and left sides with specific cellular properties improving the repolarization phase in the left atrium (LA). At tissue scale, the atrial model considers 9 types of atrial tissue with specific conduction velocity and anisotropy ratios (Crista Terminalis (CT) and the ring of the Fossa Ovalis (FO) have been also adjusted independently), and the same 21 regions with their precise fibre orientation determined from those histological studies. An important improvement has been carried out in the bridges between the Coronary Sinus (CS) and the LA since clinical and histological studies have shown the relevance of the striated myocardial muscles along its sleeve, connecting CS with the LA myocardium [38,39]. In that sense, the longitudinal conductivity on the first two proximal bridges (0,0060 *S*/*cm* ∙ *pF*) has been increased 7,5 times with respect to the four distal junctions (0,0008 *S*/*cm* ∙ *pF*) to allow the wavefront to run through these connections and reproduce a more realistic CS-LA wavefront propagation [24].

The torso model [23] has been re-meshed to increase the density of nodes in the region that overlaps the atrial model, improving the interpolation from transmembrane voltages computed at atrial scale to the finite-element tetrahedron model of the torso and then the coupling among both models. The resulting volumetric torso mesh has 254.976 nodes and 1.554.255 tetrahedral elements with a spatial resolution ranging from 0,5 mm on the atrial region to 5,8 mm on the torso surface, and divided in the same 7 regions (atria, lung, bone, liver, ventricle, blood, and general torso) with the same tissue properties as those defined in [23].

### Biophysical simulations of focal atrial tachycardia

**Fig 1:**
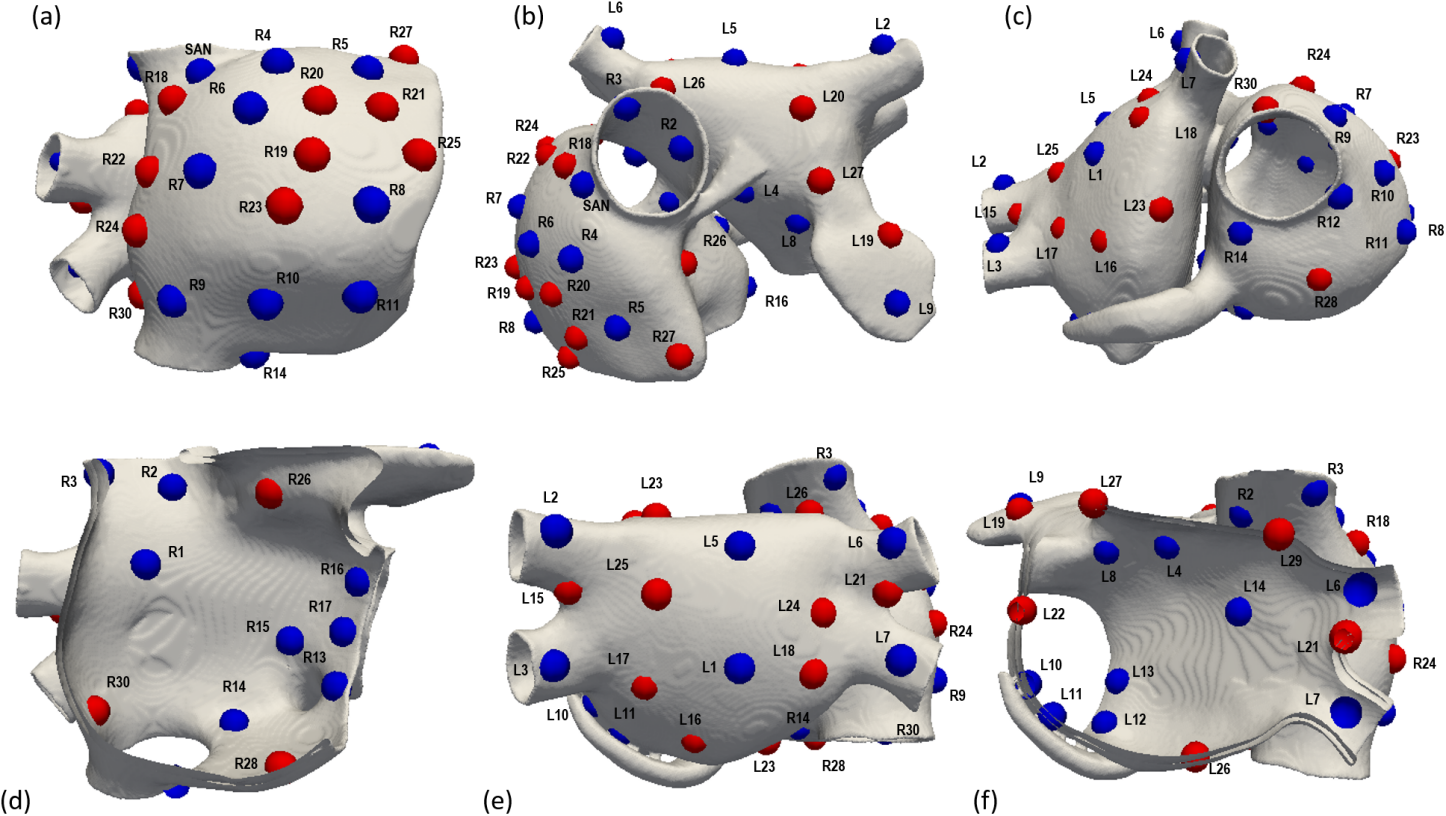
Atrial model and localization of the ectopic foci. Localization of the 58 ectopic focus sites (including the SAN) shown from six different views of the atria. Blue points labelled as R1 to R17 and L1 to L14 are the ectopic sites belonging to the validation set. Red points labelled as R18 to R30 and L15 to L27 represent the additional ectopic sites to cover the atrial walls.

First clinical studies that performed ectopic pacing in humans [1–3] stimulated in only 5–9 atrial sites: right inferior and superior and left superior pulmonary veins (RIPV, RSPV, LSPV), right atrial appendage (RAA), free right and left atrial walls, CS, left atrial appendage (LAA) and inferior vena cava (IVC). Afterwards, the number of sites was increased up to 34 divided into both atria [8–10] providing experimental patterns highly useful to validate computational simulations. However, the most recent studies [14–16,19,20] have not increased the number of the triggering sites and still stimulate in well-known locations: CT, BB, pectinate muscles (PM), atrio-ventricular ring (AVR), superior vena cava (SVC), IVC, CS ostium, RAA, RPV, LPV, mitral valve (MV), LAA, CS, and RA and LA septum. Since more accurate information on the specific trigger originating atrial tachycardia is needed, our atrial model was paced at the sinoatrial node (SAN), and at 57 distributed transmural ectopic foci (one at a time), located along the right (RA) and left atria (LA). A set of 17 sites on RA (R1 to R17) and 14 sites of LA (L1 to L14) were defined (see blue sites in Fig 1) following experimental studies [8,10] with the main aim of validating the results of the present work (validation set). These clinical studies provided a database of individual paced P-wave integral maps computed from 22 patients with normal cardiac anatomy. A first validation phase was then carried out by comparing the experimental and simulated patterns produced by each of these 31 atrial sites in terms of positive and negative areas, pathway described by the zero crossing line between both areas and location of the maxima and minima values. The additional 26 sites (13 on each atrium) were selected to cover the whole RA (R18 to R30) and LA (L15 to L27) walls and to increase the size of the training set that will feed in the machine-learning algorithms for ectopic foci location (see red sites in Fig 1). A second validation phase was performed by analysing and comparing the morphology of experimental P-wave precordial leads registered by stimulating in different sites on the RA and LA [14] with our simulated precordial P-waves computed by stimulating at the same sites. The specific anatomical sites are summarized in supporting information S1 Table.

The electrical propagation in the atria was computed by solving the reaction-diffusion mono-domain Eqs (1) and (2) with the finite element method [40]. Extracellular potentials (*V*_*e*_) were computed by an approximation of the bidomain approach. For this purpose, we firstly interpolated the transmembrane potentials (*V)* obtained in the atrial hexahedral mesh to nodes in the tetrahedral torso mesh overlapping the atrial region. Secondly, the extracellular potential was computed by solving the passive term of the bidomain model [25,41] Eq (3), using the finite elements method and Dirichlet and Neumann boundary conditions [42]

$$\nabla ∙(D\nabla V)=C_{m}∙\frac{\partial V}{\partial t}+I_{ion}\;in\;\Omega_{H}$$ (1)

$$n∙(D\nabla V)=0\;in\;{\partial \Omega }_{H}$$ (2)

$$\nabla ∙(D_{i}\nabla V)+\nabla ∙({(D}_{i}+D_{e})\nabla V_{e})=0$$ (3)

where *D* is the equivalent conductivity tensor, *D*_*i*_ and *D*_*e*_ are the volume-averaged conductivity tensors of the intra- and extracellular domains [23,43], *I*_*ion*_ is the transmembrane ionic current that depends on the cellular model, *C*_*m*_ is the membrane capacitance, and *Ω*_*H*_ is the heart domain.

The stimulation protocol consisted of an initial phase in which 20 continuous beats with a basic cycle length (BCL) of 500ms were applied to the SAN area (1180 equidistant nodes with a spatial resolution of 300 μm and a total volume of 17,8 mm^3^) to stabilize the whole 3D atrial electrophysiological properties. Following, from the steady-state conditions, a simulation was performed for each ectopic focus by delivering a single square pulse with 2 ms of duration and 28 pA/pF of amplitude on a circular area comprising an average of 542 equidistant nodes with the same spatial resolution and an average volume of 6,8 mm^3^. Since we did not include electrical remodelling, the shortest APD for our model was 170 ms (MV), which is longer than the whole atrial activation. This fact warranties that a second ectopic beat cannot start an activation sequence until the whole atria is depolarized. Therefore, the BCL of the ectopic focus will not change the BSPiM.

### Body surface potential and P-wave integral maps

**Fig 2:**
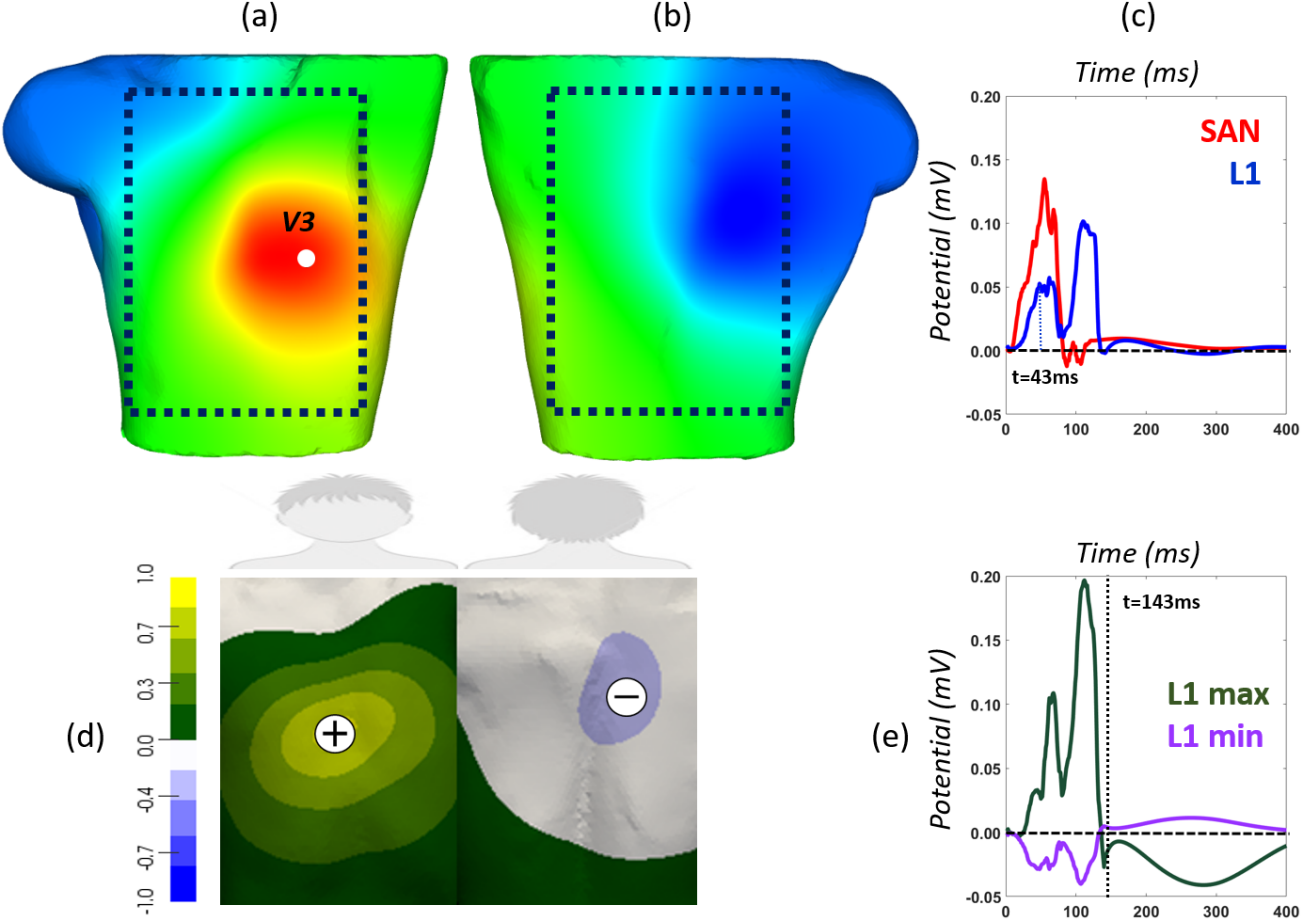
Simulated BSPMs and integral map (BSPiM) computed at the torso surface. Torso model (including the atria and the additional regions) showing an example of the frontal (a) and rear (b) views of the Body Surface Potential Map (BSPM) at time t = 43 ms, applying the ectopic beat at position L1. The dotted square represents the approximate area of the frontal and rear surface where electrographic signals may provide useful information; (c) morphology of the P-wave (potential versus time) registered at the precordial lead V3 (highlighted as a white sphere in (a) in sinus rhythm (red line) and when stimulating ectopic L1 (blue line); d) Characteristic P-wave integral map (BSPiM) (left). Greenish to yellowish area represents positive P-waves registered at any lead of this area (see green P-wave line in panel (e) registered at the position with maximum integral value), and greyish to blueish region means negative P-waves (see purple P-wave line in panel (e) registered at the position with the minimum integral value); e) Dashed horizontal baseline represents the division between positive and negative potentials. Dotted vertical line defines the duration of the P-wave defined as the latest atrial depolarization time for each simulation (t = 143 ms in the case of L1).

Simulated BSPMs were registered at the torso surface mesh (14.157 virtual leads) for each ectopic focus as shown in Fig 2A and 2B (BSPM at time t = 43 ms computed by stimulating at L1 location, see Fig 1). The dotted square encircles the region of interest, i.e., the frontal and rear regions where electrodes may be placed to register relevant P-wave electrocardiographic signals as those shown in Fig 2C. In this panel, blue line corresponds to the P-wave signal registered at the precordial lead V3 (highlighted as a white point in panel a) when the ectopic focus is paced at L1 location, and red line refers to the P-wave registered at the same lead when stimulating at the sinoatrial node (SAN). Differences between both waves were expected since principal wavefronts direction change depending on the triggering site.

Several indicators (maxima and minima values of P-wave, root mean square RMS, area under the P-wave and the product between RMS and the P-wave integral) derived from BSPM and P-waves have been considered to find out the biomarker or feature that best clusters and predicts the ectopic foci origin, using machine-learning techniques (not shown). After interpreting the information unveiled by each indicator, we choose the P-wave integral map. This integral map represents the area under the P-wave, between the zero line and the curve outlined from the P-wave onset to its offset. These two fiducial points are defined as the time at which the atrial depolarization starts from the ectopic focus and the time at which the latest atrial node is already depolarized, respectively. The body surface P-wave integral maps (BSPiM) are then displayed as a static body surface map that summarizes P-wave signals recorded at each lead. Fig 2D shows the BSPiM computed from the simulated BSPM corresponding to the ectopic focus placed at site L1. Normalized maximum and minimum BSPiM values are indicated on the map with symbols plus and minus, and the corresponding P-waves registered at V3 lead are shown in Fig 2E. The position of these extrema is relevant since they summarize the resultant direction of the electrical dipole.

**Fig 3:**
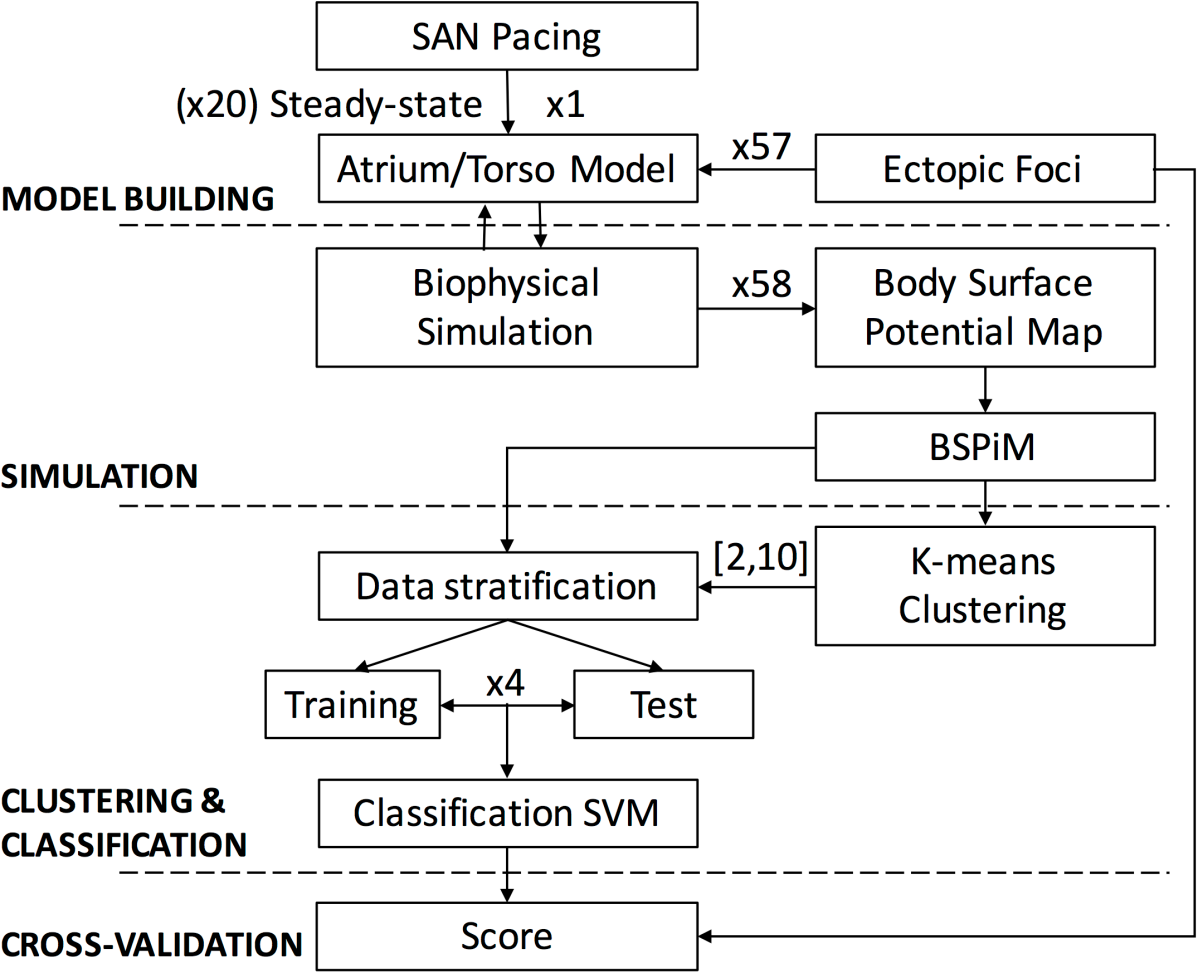
Methodological pipeline. The atrial-torso model was built up and stabilized in sinus rhythm. Afterwards, 57 new biophysical simulations were carried out stimulating at 57 additional ectopic foci in order to compute the corresponding BSPMs and BSPiMs. Machine learning techniques were used to firstly cluster these ectopic sites into spatial and well-defined atrial regions and to secondly be able to prospectively classify and predict the origin of new sites with high accuracy.

We designed a pipeline (see Fig 3) with the aim of training a system to identify the region from which an ectopic focus is triggered from the corresponding BSPiM. First, BSPiMs are calculated from the biophysical simulation of the ectopic foci. Following BSPiMs are clustered into groups, from now on ectopic clusters (EC), which number is defined by the user. Finally, the BSPiMs and their group numbers are used to train a system based on support vector machine that has to be able to classify non-observed BSPiMs into their corresponding groups.

### Clustering and classification of atrial ectopic foci from BSPiMs

We aim to find the EC, i.e. the atrial region from where an ectopic focus is paced, instead of its exact location, since the EC is more affordable and robust from a clinical perspective. To build the ECs we first compute the BSPiM corresponding to each ectopic focus and define this map as its feature vector. Therefore, our training set has 58 feature vectors (57 ectopic foci plus the sinus node activation) with a size of 14.157 torso FEM nodes each, where BSPiM is calculated. Then, we seek to validate the following hypothesis: ectopic foci localized close/near from each other in the atria will produce similar BSPiM patterns. Therefore, clustering BSPiMs will produce ECs that define non-intersected regions.

The clustering algorithms chosen were K-means [44] and EM (Expectation maximization) [45] as implemented in the python library Scikit-learn 0.18 [46]. We provided both models (K-means, EM) with the training set (R1 to R30 and L1 to L27) and split it into different ECs, i.e., k values from 2 to 10. Once the ECs were established, we validated their compactness and checked for intersections between groups.

Next, to validate the quality of the clusters obtained for different number (k) of ECs we used, as a performance oriented metric, a classification procedure. We chose a multiclass Support Vector Machine (SVM) classifier to classify the samples, i.e., the ectopic foci, considering the ECs obtained in the training phase. The aim in this second phase was to evaluate the resultant classification models using cross-validation (CV), which let us know the capability to correctly classify new samples into distinct groups. We used a stratified N-fold CV where the training set was split into N smaller sets called “folds”. Folds are selected so that the mean response value is approximately equal in all of them. This means that each fold will contain the same proportion of ectopic foci from each EC, and all the ectopic foci will be tested. The final score for the classifier is computed as the average for all the folds. In this work, we considered 4 folds, which leave 75% of samples for training and 25% of samples for testing in each fold.

## Results

### Selection of biomarkers

Among all the biomarkers used to cluster the ectopic foci, maxima and minima values of P-wave signals did not show good performance mainly because these values depend on the local signal polarity. Since root means square (RMS) of P-waves is independent of the signal polarity, it has been previously used to define those regions at the torso surface that mostly concentrate the signal [23]. The main shortcoming with this index is that important information related to the course of the wavefront direction is lost. On the other hand, as shown in Fig 2D, BSPiM overcomes the previous shortcomings considering all the information: a) the signal polarity (green to yellow areas indicate mainly positive signals while grey to purple areas mean negative P-waves); b) the regions where the energy is mostly concentrated (yellow and purple areas); and c) the atrial wavefront direction (positive P-waves are located at the frontal torso surface indicating that the atrial wavefront moves from the LA free posterior wall, L1 trigger site, towards the frontal atrial side). A combined indicator calculated by multiplying the RMS map by the integral map (BSPiM) was additionally considered (highlighting those regions with maximum energy contribution within the positive and negative integral regions). However, it did not improve results (not shown) with respect to using BSPiM alone and it was finally disregarded. Therefore, only the indicator computed as the time-integral map (BSPiM) was finally used to correlate the origin of the ectopic beat with the pattern obtained at the torso surface.

### Validation of BSPiM patterns

**Fig 4:**
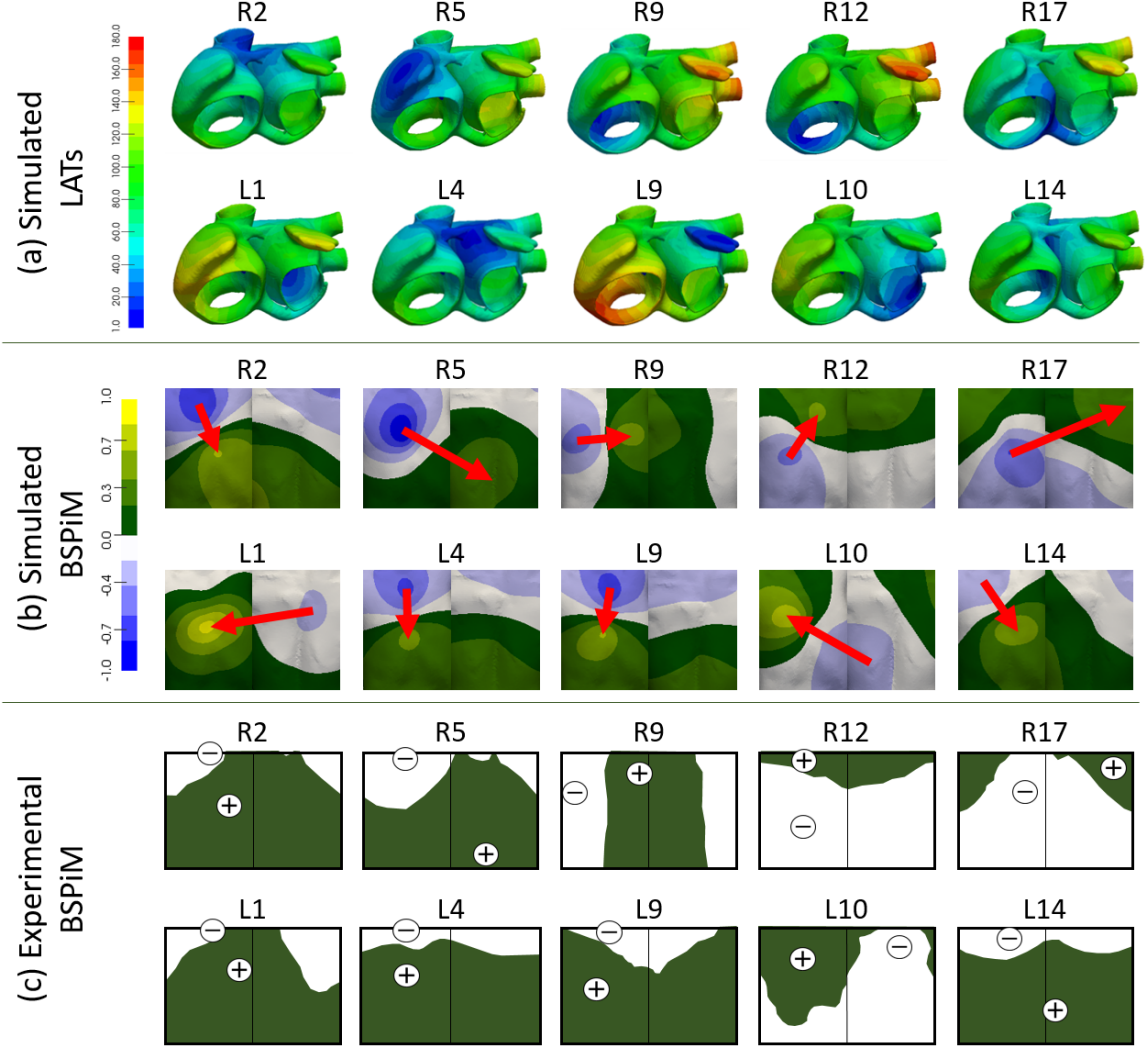
Comparison between simulated and experimental BSPiMs. (a) Simulated Local Activation Times (LATs) computed when the ectopic sites are located at different positions of the RA (2, 5, 9, 12 and 17) as defined in [8] and locations of the LA (1, 4, 9, 10 and 14) as defined in [10,11]. Bluish colour corresponds to t = 0 ms and reddish colours correspond to the latest activation time. Time is given in milliseconds; (b) Simulated normalized BSPiM computed at the 14.157 nodes of the torso surface for each ectopic focus. Bluish colours correspond to the most negative while yellowish correspond to the most positive integral values. Red arrows represent the position of the minima towards maxima integral values; (c) Experimental integral patterns reproduced from the originals published by SippensGroenewegen et al in [8–11]. White colour means negative integrals (equivalent to the blueish range for the simulated BSPiM) while green colour means positive integrals (equivalent to the greenish to yellowish range for the simulated BSPiM).

The full atrial-torso model was firstly validated by comparing the simulated BSPiMs validation set (R1 to R17 and L1 to L14) to equivalent experimental activations measured by SippensGroenewegen et al [8–10]. These clinical studies describe the use of BSPiM as a non-invasive method to identify the region of atrial tachycardia origin prior to catheter ablation using 62-lead sites superimposed over the human torso and a dataset of individual paced P-wave integral maps in 22 patients with normal cardiac anatomy. Their main result was a database consisting of 34 mean paced P-wave integral maps, 17 from each atrium. Fig 4A shows the local activation times (LATs) obtained by stimulating the atrial model at different locations of the RA (R2, R5, R9, R12 and R17) and LA (L1, L4, L9, L10 and L14). For all the remaining simulations belonging to the validation set, see supporting information S1A Fig. The total atrial activation time for simulations activated by ectopic foci localized in the RA ranges from 111 ms (R2, placed at the medial wall of the SVC) to 180 ms (R11, placed at the lateral lower wall of RA). As expected, ectopic foci at RA produce wavefronts moving leftwards and entering the LA through the BB, FO and CS. However, the movement is rightwards when the ectopic foci are localized in the LA. In this last case, the activation times range from 111 ms (L4, placed in the left side of the BB) to 173 ms (L9, placed in the LAA). It is worth highlighting how the architecture of the striated myocardial muscle connections between CS and LA strongly affects the main entrance pathway for the depolarization wavefront initiated in the low LA. Proof thereof is the fact that these bridges allow the electrical wavefront to enter the RA through the CS and depolarize both atria upwards.

For each simulated ectopic activation, the normalized BSPiM was computed at the torso surface as displayed in Fig 4B (for all the others sites belonging to the validation set, see supporting information S1B Fig). These simulated BSPiMs were analysed and compared to the experimental integral patterns published in [8–10] and reproduced in Fig 4C. When comparing each pair of simulated-experimental patterns, it can be noticed that both match in terms of distribution of positive and negative values of the P-wave integral as well as in the position of the maxima and minima values. In the RA, three different patterns can be observed depending on the direction of the vector that links the minimum and maximum values (see Fig 4B, upper row and S1B Fig of supporting information for all the others sites): i) downwards for ectopic foci originated at the upper face of this atrium (R2 and R5), ii) upwards for ectopic foci placed near the tricuspid valve (R12 and R17), and iii) left-sided for ectopic foci originated at the lower atrial backside (R9). The same trend is observed in the experimental patterns that even match the position of the maxima and minima values of the integral (see Fig 4C, upper row and S1C Fig of supporting information for all the others sites). In the case of ectopic sites localized in the LA (see Fig 4B, lower row), atrial activation is mostly influenced by the conduction pathways between both atria. Thus, when the wavefront first arrives to the RA through the BB and FO (L4, L9 and L14), the simulated BSPiMs are positively signed on the medium and lower torso. However, when the wavefront enters the RA through the CS (L1 and more visibly in L10) the BSPiM changes [24] and mainly shows positive values on the frontal torso. Similar patterns and location of the maxima and minima values are again observed experimentally (see Fig 4C, lower row), except in the case of L1 where the most negative value produced by our simulation is shifted from the frontal to the rear side on the torso model. When analysing the position of the L1 maxima and minima values, it can be observed that the position of the maximum positive is correct while its minimum negative value is moved from the expected frontal upper position to the posterior medium area. If we try to find other ectopic sites that produce the most negative value at that frontal upper area, but keeping a similar position for the positive maximum, we might find R2, R26, L4, L15, L19, L20, L22, L25, L26 or L27. These sites are shifted leftmost and a bit further up with respect L1. Probably placing L1 nearer L25, the position of its extrema values would be improved while keeping its BSPiM. Despite this case, the overall distribution of the integral values across the torso surface shows similar patterns for the whole validation set of simulated and experimental BSPiM shown in the supporting information S1 Fig.

**Fig 5:**
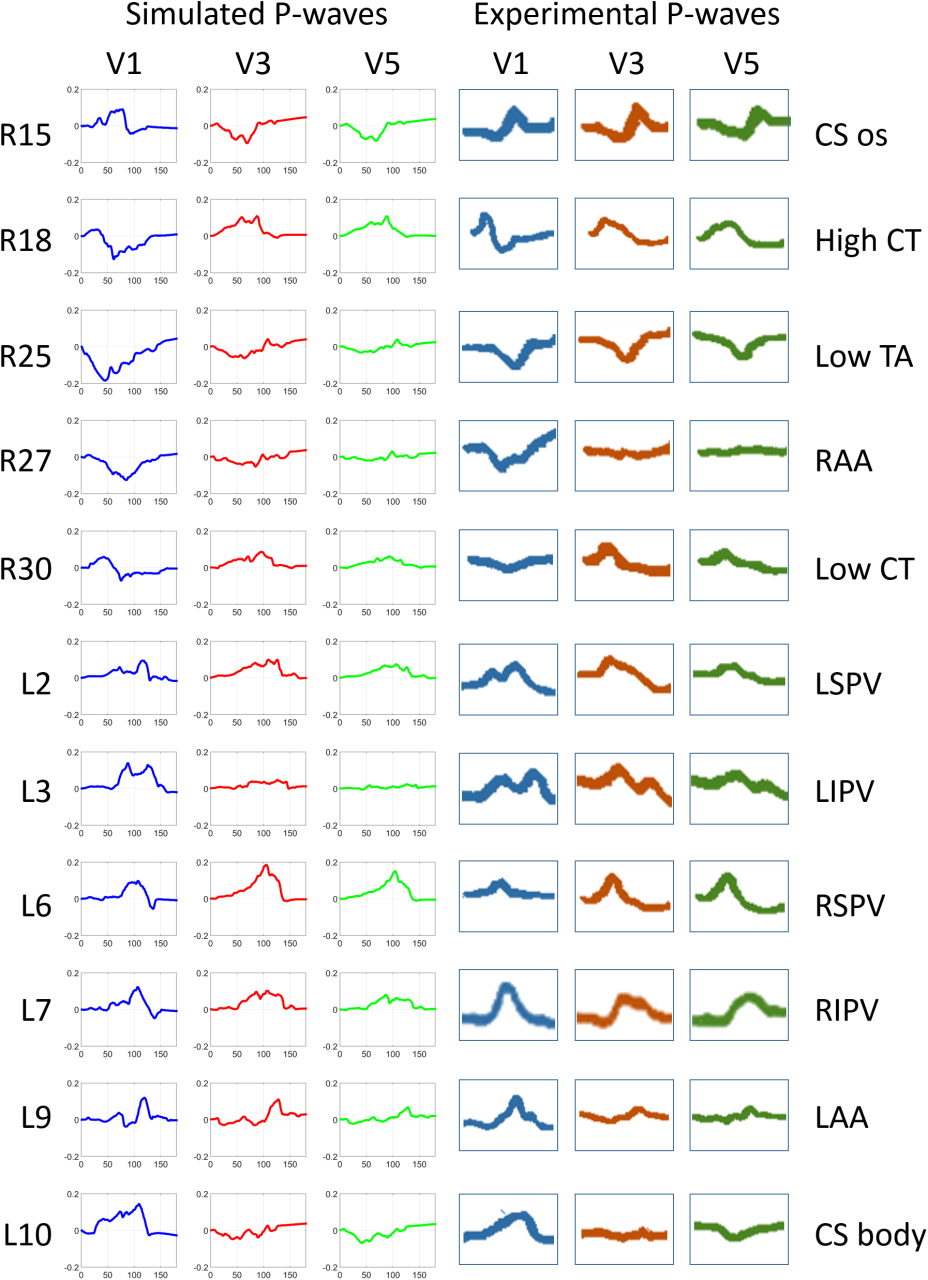
P-wave morphology registered in precordial leads produced by simulations (left panel) and by experimental activations (right panel). Left panel: P-wave registered at V1 (blue), V3 (red) and V5 (green) from the RA simulations 15, 18, 25, 27 and 30 and from the LA simulations 2, 3, 6, 7, 9 and 10; Right panel: P-waves reproduced from the experimental recordings by Kistler et al in [14] at the same sites as used to simulate. Experimental stimulated regions: ostium and body of the CS (CS os and CS body), higher and lower CT (High and Low CT), lower region of the TA (Low TA), right and left atrial appendages (RAA and LAA), left and right and superior and inferior PVs (LSPV, RSPV, LIPV, RIPV).

The second validation test for the model consisted of analysing and comparing the morphology of the P-wave signals in specific leads as carried out experimentally by Kistler et al [14], who also stimulated in different sites on the RA and LA. Fig 5 compares simulated (left panel) vs experimental (right panel) leads V1, V3 and V5 registered when the ectopic foci were placed in the CS (ostium and body), CT (low and high), tricuspid annulus (TA), atrial appendages (RAA and LAA) and pulmonary veins (LSPV, LIPV, RSPV and RIPV). Among the whole dataset of 57 in-silico ectopic foci plus the SAN, those localized nearer the experimental sites were chosen in order to compare equivalent simulated-experimental P-wave morphologies. In general, the P-wave polarity and morphology closely followed similar trends in the experimental and simulated domains for the three leads and for all the anatomic sites tested. Examples of this similar behaviour for the simulated-experimental pairs are: i) the pair R27-RAA shows a negative V1 while V3 and V5 are isoelectric; ii) the pair L3-LIPV shows a noteworthy morphology in V1, that is a characteristic double peak, also reproduced by the equivalent simulation L3; iii) in the case of L30-CS body, V1 is positively signed in both, simulated and experimental leads, while V3 is isoelectric and V5 slightly negative in both domains. All the remaining simulated P-waves registered at the same leads for the 57 ectopic foci and the SAN are shown in supporting information S2 Fig. In general, the morphology described by all the P-waves at the three leads, and for all the simulations, is coherent with the position of the corresponding ectopic focus. Besides, sites placed around the same atrial region also produce P-waves with similar trend.

### Clustering atrial ectopic sites from BSPiM patterns

**Fig 6:**
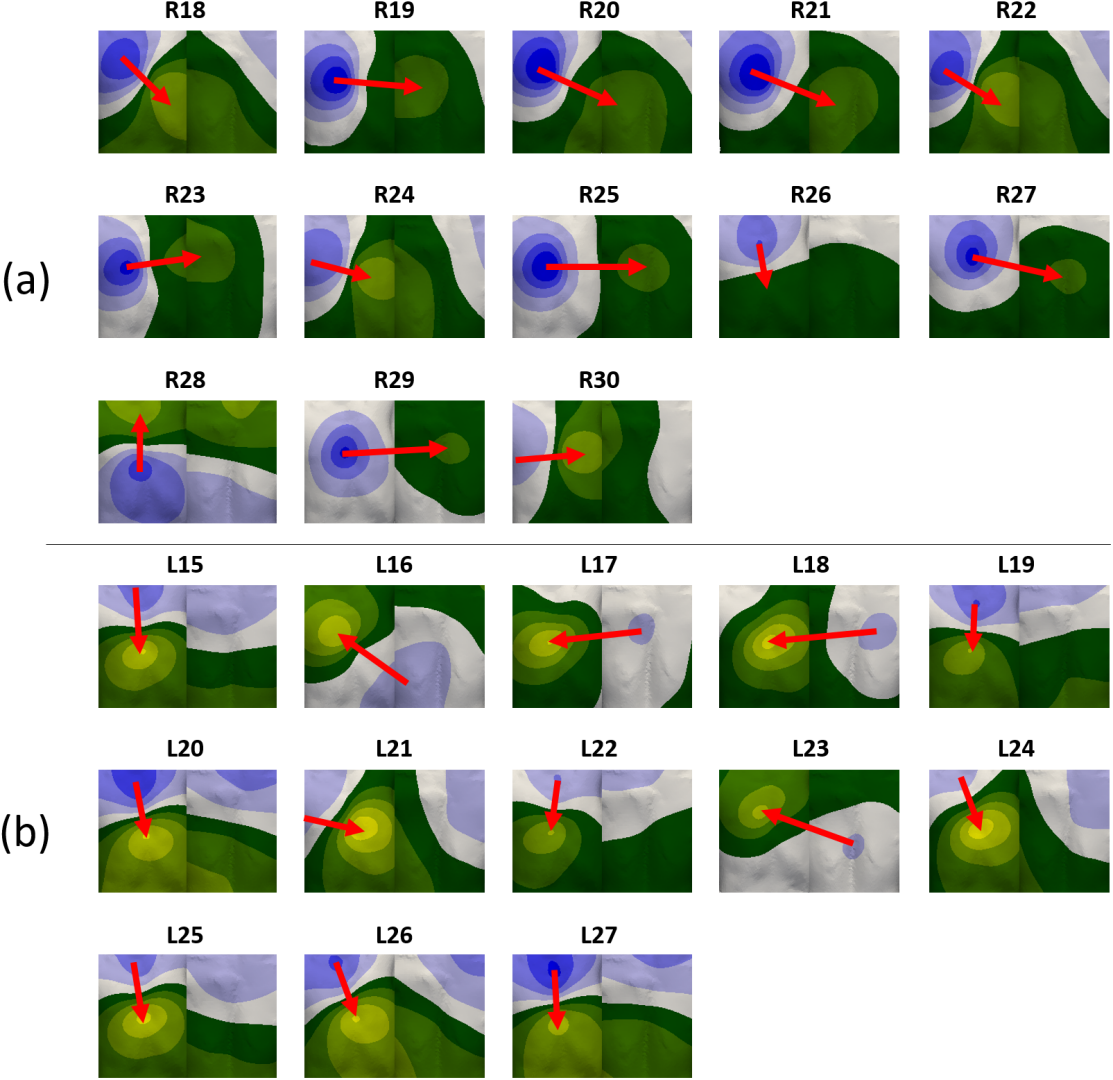
Simulated normalized body surface P-wave integral maps (BSPiM). BSPiMs have been computed at the torso surface for the 26 additional ectopic foci stimulated at a) RA (R18 to R30), and b) LA (L15 to L27). Normalization was carried out based on the maximum range of P-wave integral considering the 58 different BSPiMs.

We checked whether our initial hypothesis, which proposed that close/near ectopic foci in the atria should produce similar BSPiMs, was true. For this purpose, we used the whole dataset of 58 triggers aiming at covering the whole atrial tissue. The 26 additional BSPiMs were computed making up a database with 58 BSPiMs (including those belonging to the validation set and the SAN stimulation). Fig 6 displays the patterns corresponding to the additional 26 ectopic foci. The same three kinds of patterns can be observed for the sites localized in the RA (Fig 6A): i) downwards (R18, R20, R21, R22, R26 and R27) for foci originated from the upper side of this atrium, ii) upwards (R28) for ectopic foci placed near the tricuspid valve, and iii) leftwards (R19, R23, R24, R25, R29 and R30) for ectopic foci originated in the medial lower side. In the case of ectopic sites in the LA (Fig 6B) these patterns show again distributions similar to the ones showed in Fig 4A: i) downwards (L15, L19, L20, L21, L22, L24, L25, L26 and L27) for ectopic sites localized in the upper half of the LA forcing the wavefront to enter the RA through the BB and FO, and ii) rightwards (L16, L17, L18 and L23) indicating the atrial depolarization wavefront enters the RA through the CS from sites placed in the lower rearward of the LA.

**Fig 7:**
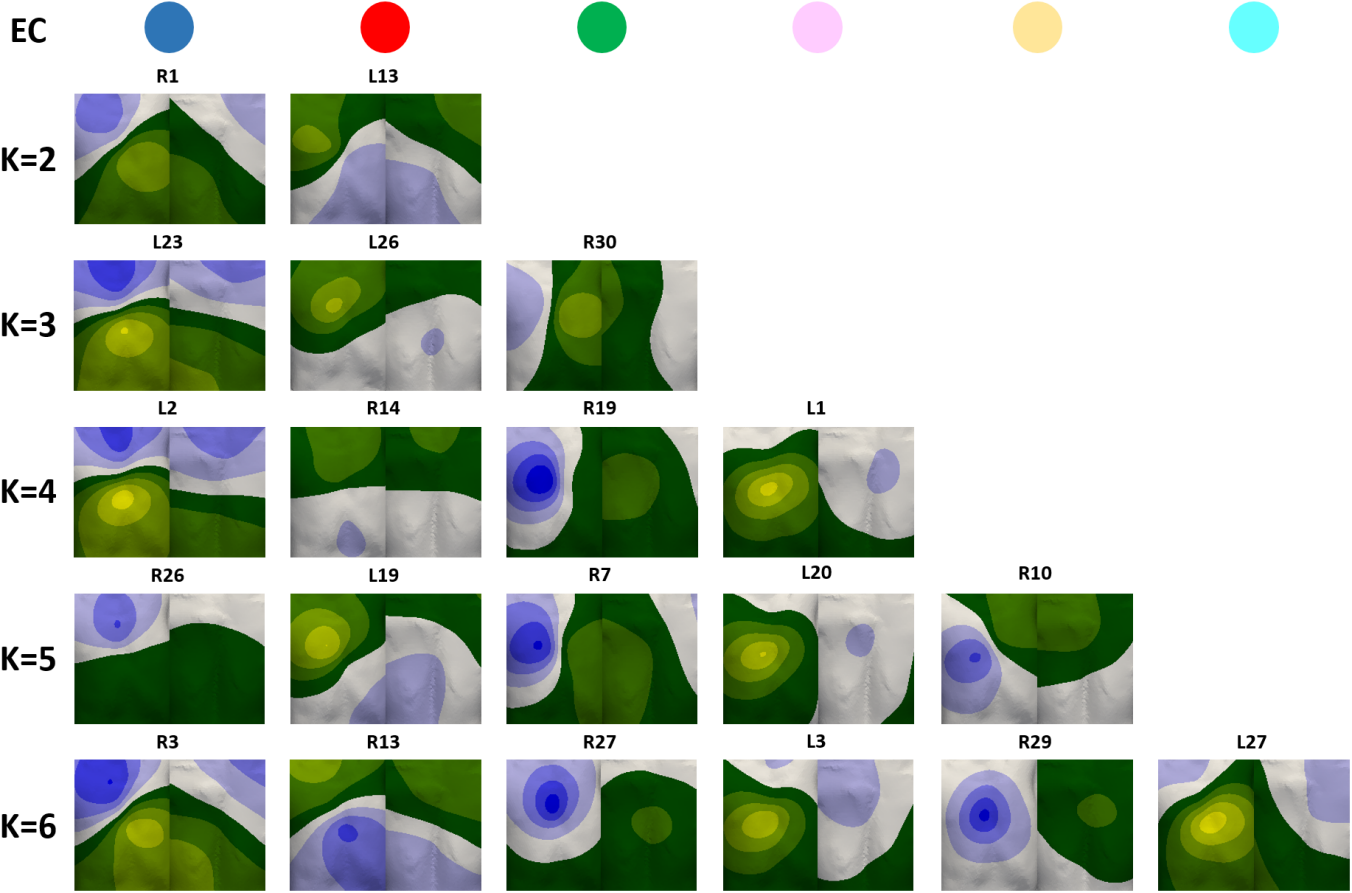
Exemplary BSPiMs included in ECs for different number of clusters (k) from 2 to 6. A single pattern belonging to each coloured class for each k is displayed.

**Fig 8:**
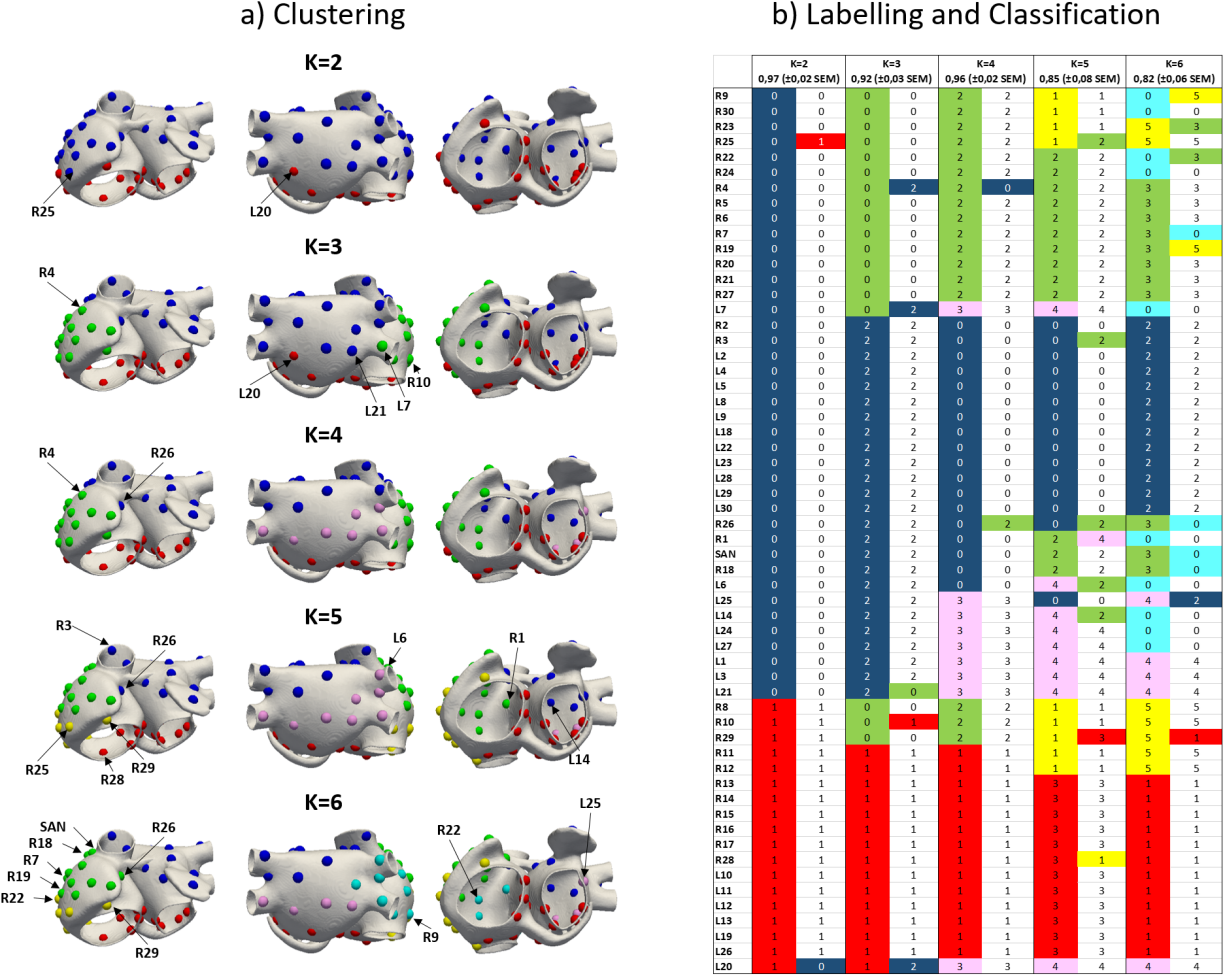
Clustering and classification of BSPiMs. a) Clustering of the 57 patterns of BSPiM (30 from RA and 27 from LA) plus the SAN using the K-means algorithm on the torso surface nodes where K is the number of pre-defined ECs. For each K, all the foci belonging to the same EC have the same colour. b) For each K, left column shows the colour and number of each EC resulting from the clustering step (for example blue-k2-0 refers to blue EC, equivalent to class 0, when k = 2) while right column shows the classification results identifying the ectopic site that is not well classified with the colour of the correct group, to which it really belongs.

The dataset of BSPiMs was then clustered into ECs by K-means and EM algorithms, in order to group ectopic foci that produce statistically similar and well-defined BSPiMs. Both algorithms produced almost the same results for all the ECs. Fig 7 shows an exemplary BSPiM from each EC (upper row with coloured circles corresponds to individual cluster IDs) for each k (number of ECs in which the whole database of 58 BSPiMs is split). Additionally, Fig 8A displays these clusters, computed by using the K-means algorithm for different values of k (all the foci belonging to the same group have the same colour ID previously defined by the coloured circles in Fig 7).

For k = 2 the dataset is divided in two different groups. The first one shows the positive signed integral values in the lower and leftmost side of the frontal and rear torso (e.g. R1, Fig 7 blue EC), and corresponds to ectopic sites localized in the upper part of both atria (blue points in Fig 8A corresponding to blue-k2-0 in Fig 8B). The other cluster shows the opposite, i.e., positive values at the upper and rightmost side of the torso (e.g. L13, Fig 7 red EC) and corresponds to ectopic sites in the region closer to the tricuspid and mitral annulus (red points in Fig 8A corresponding to red-k2-1 in Fig 8B).

For k = 3, BSPiMs associated with blue and red ECs remain almost unchanged with respect to patterns with k = 2 (Fig 7). The new green EC shows positive integral values all along the left side of the torso on both, the front and back (e.g. R30). The fact is that, as depicted in Fig 8, blue-k2-0 mainly divides itself into two classes giving rise to the clusters in the right (green-k3-0) and left (blue-k3-2) atria including the septum and the superior vena cava. Interestingly, red-k3-1 previously associated with the lower side of both atria only suffers a very slight movement of the borders between the three classes. If we continue increasing the number of groups to k = 4, the green EC with k = 3 splits up giving rise to a pattern with positive integral values further backwards as is the case of R19 and new (pink) EC where the positive values are mainly computed at the frontal torso surface. In the atria (Fig 8A), the new yellow class when k = 5 is the result of splitting the green and red ECs (k = 4) giving rise to this new EC localized in the lower right side of the RA. Finally, when k = 6 class subdivision starts mixing up ectopic foci from previous pink, green and yellow groups (k = 5) and the identification of the area is slightly difficult as can be seen in the lower row of Fig 8A. When the number of classes (k) increases beyond 6, it is difficult to clearly assign some of the BSPiMs into ECs since the number of ectopic foci within each class starts decreasing significantly (see for example the 5 sites within the pink EC and the 7 sites within the yellow EC) and the size of the spatial region is considerable reduced not allowing to clearly differentiate P-wave integral patterns among clusters.

### Prospective classification of ectopic foci

Considering the ECs defined during the previous clustering phase, we study the accuracy of a learning algorithm to classify ectopic foci into the ECs. We use support vector machines (SVM) with a radial basis function to learn the BSPiM patterns and their associated ECs. Following, the classification accuracy is determined by a 4-folds stratified cross-validation (CV). Results are shown in Fig 8B. For each fold, around 13–17 ectopic foci with representatives of all classes are used for testing the system, and the remaining for training the SVM. High values of accuracy imply that the majority of the ectopic sites are properly classified, while lower values highlight that some sites are assigned to clusters to which they do not really belong.

For k = 2, only 2 out of 58 sites (R25 and L20 highlighted in Fig 8) were wrongly classified and the final accuracy of the classifier was 97%. As can be observed in Fig 8A, these two foci are located at the border between both ECs so they might belong to any of the classes. When k = 3, there are 5 out of 58 sites at the border between ECs wrongly classified (R4, R10, L7, L20 and L21) resulting in a lower accuracy (92%). For k = 4, the ectopic sites classified incorrectly are R4 and R26 bringing again the accuracy to higher values (96%). For these previous clustering and classification results, and mainly when k = 2 and k = 4, the results in terms of accuracy can be considered excellent (higher than 95%). However, when k = 5 (and 6), the accuracy decreases below 90%, what means that more ectopic sites are being wrongly classified. As k increases, the number of ectopic foci in each EC decreases, the classifier is not able to discriminate BSPiMs and the accuracy falls under 80%.

Given our training set of 58 BSPiM, 4 ECs is the optimum number of clusters, dividing the atria into four regions as depicted in Fig 8A and 8B: i) top left (blue-k4-0) including superior vena cava, Bachmann’s bundle, left superior wall, left appendage, superior side of the left posterior wall, superior pulmonary veins, ii) bottom right and left (red-k4-1) mainly including tricuspid and mitral valves, inferior vena cava and isthmus, iii) further right (green-k4-2) including right lateral wall and appendage and the intercaval bundle, and iv) bottom back left (pink-k4-3) including inferior pulmonary veins and lower side of the left posterior wall.

## Discussion

In this paper, we present an enhanced multi-scale atrial-torso model that allows generating BSPMs and validating BSPiMs, which are exploited by machine learning techniques to associate BSPM-derived biomarkers with the localization of atrial foci in cases of focal atrial tachycardia. Given a prospective BSPiM, our computational pipeline is able to predict from which atrial region was the focal atrial tachycardia triggered with an accuracy over 95% (considering between 2 and 4 regions). We additionally provide a database with 58 simulated BSPiM patterns with 14.157 samples computed by placing the ectopic foci across both atria.

### Computational modelling and validation

In the 3D atrial model, the adjustment of the connections of the proximal and distal CS-LA bridges have improved the realism of wavefront propagation between both regions, compared to our previous model [23]. In fact, the majority of the existing atrial models [47–55] do not include the CS and only a few [36,56] considered this region. The effect that these bridges have on the P-wave morphology and the P-wave integral patterns is remarkable in some configurations. For instance, they fully determine the pathway followed by the depolarization wavefront when an ectopic focus is localized in the low LA. Proof thereof is the fact that when the bridges are blocked, the wavefront enters the RA through the BB and FO depolarizing the LA upwards and the RA downwards affecting the morphology of the P-wave and therefore altering the BSPiM [24]. However, when these junctions are activated, both atria depolarized upwards. The BSPiMs computed with this last configuration [24,38,39] are very much alike the experimental ones by Sippensgroenewegen [10,11] and helped us to validate the model.

The two-step validation process compares first our simulated validation set of 31 BSPiMs, concerning ectopic triggers placed in R1 to R17 and L1 to L14, with experimental integral patterns [8–11] and afterwards compares some of our P-wave morphologies with experimental signals [14]. The results confirmed that our atrial-torso model is accurate since simulated patterns match the experimental measurements. This allowed us to increase the number of ectopic foci covering the whole atrial walls and build up a database of 58 BSPiMs to feed in the machine-learning algorithms for ectopic foci localization.

### Clustering and classification of BSPiM

Among the BSPM-derived biomarkers tested to non-invasively cluster atrial ectopic sites, the BSPiM provided the best results in terms of localizing ectopic atrial triggers. This biomarker takes into account the main characteristics of the P-wave signal and atrial depolarization: morphology, atrial depolarization wavefront and potential distribution towards the torso surface. The clusters obtained by using K-means (or EM) algorithm were not intersected and defined anatomical atrial regions so that they could be useful to guide clinicians during invasive interventions like radiofrequency ablation of ectopic sites. When the number of ECs considered is between 2 and 4, the clusters of BSPiMs are clearly differentiable. As the number of ECs increases, the new classes do not provide clearly distinguishable patterns probably because the number of ectopic sites within those new classes is very low.

Beyond the good performance of the clustering process, the results obtained from the classification phase are also very promising. In this regard, the wrongly classified ectopic sites when the number of ECs is between 2 and 4 are always localized at the borders between classes and the accuracy in classifying raises up to 97%. However, as the number of ECs increases, the classification errors also increase thereby reducing the accuracy to values below 90%. Technically, this may be due again to the fact that the number of ectopic sites within each cluster is dramatically reduced (sometimes below 6 sites) and the classifier, which only uses 75% of available sample for training, has not enough information to correctly assign the patterns to the pre-defined clusters. As can be observed in Fig 8B, those clusters with fewer samples show larger misclassification rates. This could be overcome increasing the database of feature vectors mapping the atria with additional trigger ectopic sites. We will extend our database in the future to test whether our methodology can improve the classification rates for larger number of clusters.

### Previous studies

All the experimental studies based on the analysis of the ECG and BSPM [1–7,13] highlighted the need to localize the origin of the ectopic foci non-invasively and provided a first-hand knowledge of the changes on P-wave characteristics and potential distribution on the torso surface when the atrial tachycardia origins at different atrial regions. These studies drew interesting conclusions: i) P-wave morphology in lead V1 was the most informative for the diagnosis of left atrial rhythms [1]; ii) when depolarization starts from a focus within the RA and coronary sinus, the resultant P-waves consistently assumed an orientation determined by the site of stimulation [3]; and iii) P-wave dipole evolution may correlate the dipole trajectory with specific RA-paced regions [13], but when the ectopic site is localized in the LA, the poles changed its position and amplitude very fast giving rise to big jumps forward and backwards. However, several shortcomings prevented authors from clearly relating torso surface electrical phenomena to atrial myocardial events: a) the techniques used were not much accurate mainly with P-waves with low amplitudes or isoelectric in surface leads; b) it was always emphasized the necessity of using intracardiac intervention to measure the atrial depolarization times; and c) the isopotential map characterized by a single maximum and/or a single minimum did not show a direct correlation with the origin of the atrial ectopic foci.

Our results already confirm all these experimental conclusions. In this way, patterns produced by ectopic sites localized in the RA are very much clear and easily clustered and classified than those produced from the LA. The main reason is that bridges from RA towards LA define a coherent movement of the wavefront always leftwards. However, the path followed by this depolarization wavefront is extremely dependent on the interatrial bridges on the opposite direction (from LA towards RA) and then patterns are completely different depending on the RA tissue in being firstly depolarized (from the BB, the FO or the CS) [24].

Short time after BSPM arose as an integral part of the mapping protocol during radiofrequency catheter ablation procedures, the use of BSPiM shed much more light on how to noninvasively determine the arrhythmogenic target region for ablation using a single beat analysis approach [8–11]. In this regard, our BSPiMs and those patterns experimentally obtained pacing at upper and lower regions of the RA showed similar trends with respect to i) the opposite positions of the maxima and minima extremes, ii) the rotation of the P-wave forces as pacing site moves downwards and iii) the movement of the zero-line contour between segments (equivalent to our ECs). Coherent results have also been obtained from our simulated and the experimental P-wave integral patterns when the ectopic triggers are localized in the LA, always dependent on the firstly-activated bridge between LA and RA. Exceptionally, BSPiMs computed when pacing sites are placed at the FO are very similar to patterns obtained by pacing at other LA sites and therefore there is no certainty about the unique origin of the ectopic focus [10]. Although these experimental studies provided 34 mean maps (17 from each atrium), authors concluded that it is complex to differentiate among those patterns localized close to each other, as it is the case, for example, of experimental R1 and R2, R7 to R9, R12 to R14, L1 to L3 or L11 to L12. This experimental inter-pattern similarity led us to conclude that our spatial ECs are much more representative of specific atrial regions responsible for triggering ectopic foci.

On the other hand, results provided by other techniques such as ECGi during experimental atrial pacing in humans [17–19] and in patients with paroxysmal or persistent atrial fibrillation [20], could determine specific triggering sites (mainly lateral RA and PVs from Non-PV) on the basis of earliest activation and localization of the potential minimum and maximum. However, the authors were not able to distinguish between, for example, upper from lower pulmonary veins, RA from LA or these two from the atrioventricular rings. Experimental ECGi presents three main shortcomings. First, it is highly dependent on the inherent atrial signal quality, as it is the case for ECG or BSPM, leading to discard many leads with low signal to noise ratio. Second, it reconstructs potentials only on the atrial epicardial surface, ignoring effects of the myocardial atrial wall. That requires an imaging study and the reconstruction of the atrial and torso 3D domains, which is highly complex and time consuming. Third, some regions remain difficult to image like interatrial septum, the left pulmonary veins and the LA appendage ridge. All this may lead to incorrect computational assumptions that can influence the reconstruction of the atrial potentials. From the analysis carried out during the present work, we can infer that P-wave integral is a more robust biomarker against low quality P-wave signals since the area under the signal is a simple mathematical computation that depends on the signal amplitude or morphology. Furthermore, the whole atrial wall is always taken into consideration to compute the forward BSPiM and then to inversely cluster and classify ectopic foci on the atrial wall.

When analysing computational approaches, there are relevant studies that have provided interesting results. In this regard, a simple decision tree algorithm was firstly constructed using experimental P-waves [14] and posteriorly used with computational models [15] for the identification of the anatomic triggering atrial sites based on the P-wave morphology. This algorithm was able to correctly identify the focus in 93% of the experimental cases while the accuracy decreased to an average of 85% (and even lower with bi-phasic P-waves) with their simulation results. Based on the morphology, authors grouped triggering sites in a) crista terminalis overlapped sometimes with right pulmonary veins, b) tricuspid annulus together with RA appendage; c) coronary sinus ostium overlapped with left septum, d) perinodal together with right septum, e) left pulmonary veins together with LA appendage, f) mitral annulus and g) CS body. Interestingly, our results show a higher accuracy above 95% without intersecting critical clusters such us those including PVs, CT or CS.

A more recent algorithm was developed [16] to obtain the triggering site of the stimulus from a 64-lead ECG system with a success rate of 93%.The authors divided both the torso and the atria in 8 quadrants and used the P-wave polarity to quantify the differences in morphology. All these computational approaches assumed however, predefined areas what might lead to a bias when interpreting the results, and found the same problems associated with bi-phasic or irregular P-waves. Our methodology overcomes this bias by using clustering algorithms that allow a first natural pooling of the BSPiMs without any initial constrain or starting point.

These approaches based on the use of single algorithms have evolved to the recent and more complex combinations between computational models and machine learning techniques. Previous studies based on machine learning techniques mainly focused on predicting ventricular pacing sites [21] and on classifying cardiac excitation patterns during atrial fibrillation on tissue patches [22]. In [21] a set of features obtained from simulated BSPM signals are learned using Kernel Ridge Regression, including: the position of the global extremum, the absolute potential of the global extremum, the sign of the global extremum, or the number of zero crossings. On the contrary, we use support vector machine regression (SVM), and choose a single feature, the BSPiM, from a set of features analysed such as maximum, minimum, root means square and integral maps. Another difference is that in [21] they try to learn and predict the full ventricular activation time, whereas we associate each BSPiM to a class, which is related to a focal ectopic site. In [22], they choose also as a learning algorithm a multiclass SVM, but to differentiate between fibrillation activation patterns and classify them into 4 groups (plane waves, ectopic focus (spherical wave), rotor (spiral wave) and block). We only work with patterns derived from focal atrial tachycardia, and therefore we cannot recognize other types of arrhythmias. The applications and goals of other works that use machine-learning techniques are very different from ours, and therefore comparing their accuracies will not provide useful information.

## Study limitations

Inter-patient variability is always a common clinical problem when diagnosing atrial arrhythmias what may prevent physicians to draw general conclusions. Even the use of computational models may lead to similar shortcomings unless they are based on proper virtual population cohorts. This fact led us to validate our simulations using a database of 34 experimental mean P-wave integrals from 22 patients. Additionally, we propose the use of the normalized BSPiM as a robust biomarker against small or local variations of the P-wave morphology. However, additional work must be done to build a larger set of representative atrial-torso models, and to check the accuracy of the machine-learning pipeline in presence of fibrotic areas or uncommon atrial morphologies. Current commercial BSPM systems are equipped with around 256 electrodes or less, which is a small number compared to the 14.157 electrodes that includes our torso surface. Additional work should be carried out to reduce the number of leads and define the optimal surface ECG electrode set in terms of number and position on the torso surface to obtain similar clustering and classification results. Having a sufficiently large number of ectopic samples for training the system, the accuracy could be improved even increasing the number of ectopic atrial regions in which the atria is divided, which could improve clinical usefulness. Finally, our methodology allows classifying prospective BSPiMs into predefined groups associated with atrial regions of different sizes. Therefore, differently to other techniques such as ECGi, we cannot provide a spatial accuracy in our predictions, since the ectopic beat could be anywhere in the region, and then the region size changes depending on the number of clusters considered.

## Conclusions

Following this methodological approach, the present study is the first in using clustering and classifying techniques to localize atrial triggering sites providing promising and useful results for physicians. In this regard, our validated atrial-torso multi-scale model has been used together with k-means clustering algorithms and classifying models based on multiclass support vector machine (SVM) providing an accuracy of up to 97%. Although the classification capability with reduced spatial information should be investigated in future studies, these promising results could encourage physicians to use the BSPiMs as non-invasive biomarker and machine learning techniques to guide, for example, the radiofrequency ablation procedure or any other invasive procedure to restore patients to sinus rhythm.

## Clinical relevance of this study/clinical implications

There is extensive literature describing the use of different algorithms using the P-wave morphology on the 12-lead ECG to identify the site of origin of focal atrial tachycardia (FAT). Although the standard 12-lead surface ECG is a useful tool for providing an initial approximation of the site of origin of FAT, it has numerous limitations. In fact, different sites of origin could yield nearly identical P-waves morphology. Moreover, the overlap in P-wave morphology reflects the limited spatial resolution of the P-wave that inevitably gives rise to prolong and even repeat or failed procedures. The combination between BSPiMs and machine learning techniques is a promising tool for the diagnosis of the tachycardia origin and, as shown here, could be useful in procedure planning before catheter ablation. The multi-scale atrial-torso model combined with machine learning techniques used in the present study enabled accurate location of the vast majority of FAT and could eventually help in a safer and more efficacious catheter ablation of the tachycardia.

## Supporting information

S1 Fig Validation of the atrial and torso models comparing the experimental activations with the simulations carried out by activating ectopic foci at the same sites. a) Local activation times (LATs) computed by activating the SAN, and the 17 RA sites (upper panel) defined in [8] and at the 14 LA sites (lower panel) defined in [10,11]. Bluish colours correspond to t = 0 ms and reddish colours correspond to the latest activation time; b) Simulated normalized BSPiM computed at the torso surface for each ectopic foci. Bluish colour means the most negative integral value and yellowish means the most positive integral value; c) Experimental integral patterns reproduced from the originals computed by SippensGroenewegen et al and published in [8–11]. White colour means negative P-waves (equivalent to the white to blue range for the simulated BSPiM) while green colour means positive P-waves (equivalent to the green to yellow range for the simulated BSPiM).
(TIF)

S2 Fig P-wave morphologies registered for the 57 ectopic sites plus de SAN. Position of the precordial leads on the torso surface and P-wave morphology registered at V1 (blue), V3 (red) and V5 (green) for the sites stimulated on RA (30 sites plus SAN) and the sites stimulated on LA (27 sites). The doted purple square, for example, identifies the P-waves registered at V1, V3 and V5 produced by the ectopic site R15. In the case of the dashed brown square, it identifies the P-waves produced by the ectopic site L26.
(TIF)

S1 Table Anatomical sites of the atrial ectopic foci. 58 ectopic foci (including the SAN) grouped by RA and LA segment locations. The first 17 and 14 sites on RA and LA respectively (31 sites in total) were placed at the same positions used in previous experimental studies [8,10]. The additional 13 sites on each atrium (26 sites in total) were randomly selected to cover the whole atrial walls.
(TIF)
