# Supplementary figures and images for: Non-invasive localization of atrial ectopic beats by using simulated body surface P-wave integral maps
Source: PLoS One. 2017 Jul 13;12(7):e0181263. doi: 10.1371/journal.pone.0181263 (PMC5509320; doi:10.1371/journal.pone.0181263)

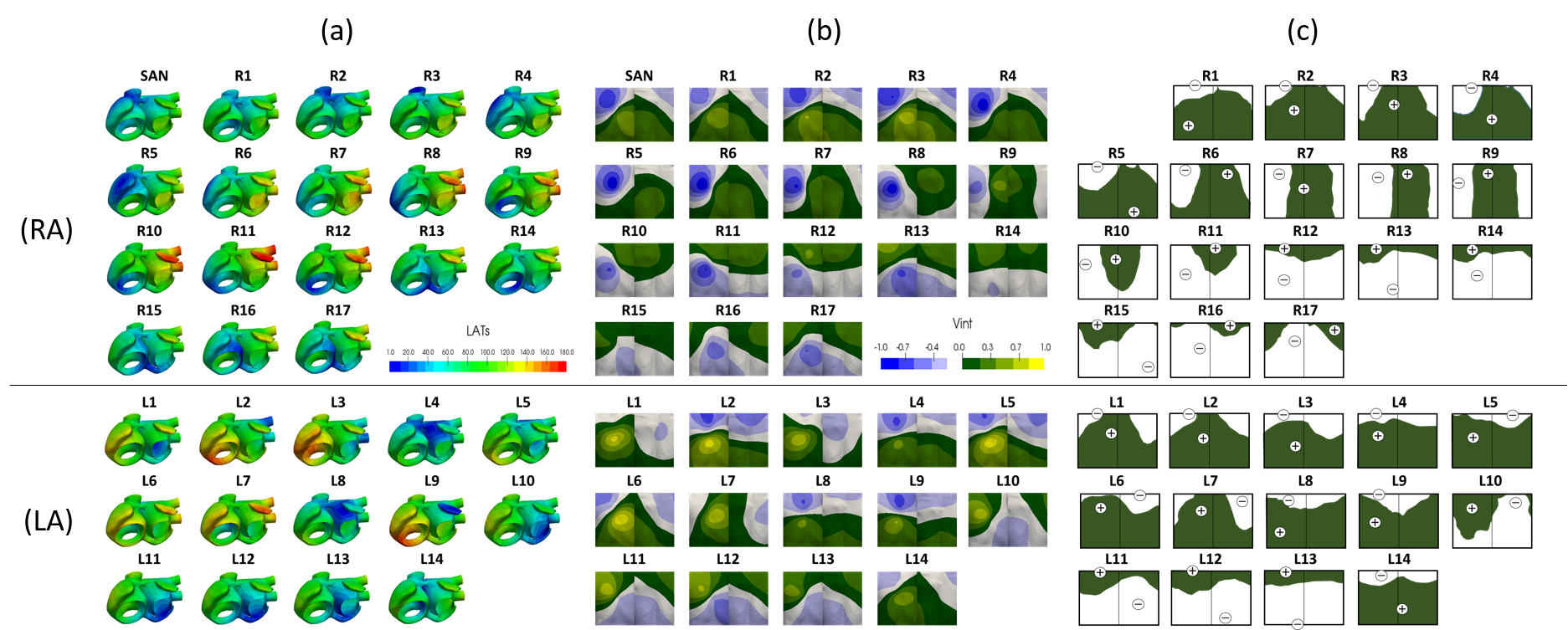

Supplement: S1 Fig — a) Local activation times (LATs) computed by activating the SAN, and the 17 RA sites (upper panel) defined in [8] and at the 14 LA sites (lower panel) defined in [10,11]. Bluish colours correspond to t = 0 ms and reddish colours correspond to the latest activation time; b) Simulated normalized BSPiM computed at the torso surface for each ectopic foci. Bluish colour means the most negative integral value and yellowish means the most positive integral value; c) Experimental integral patterns reproduced from the originals computed by SippensGroenewegen et al and published in [8–11]. White colour means negative P-waves (equivalent to the white to blue range for the simulated BSPiM) while green colour means positive P-waves (equivalent to the green to yellow range for the simulated BSPiM). (TIF) [file pone.0181263.s001.tif]

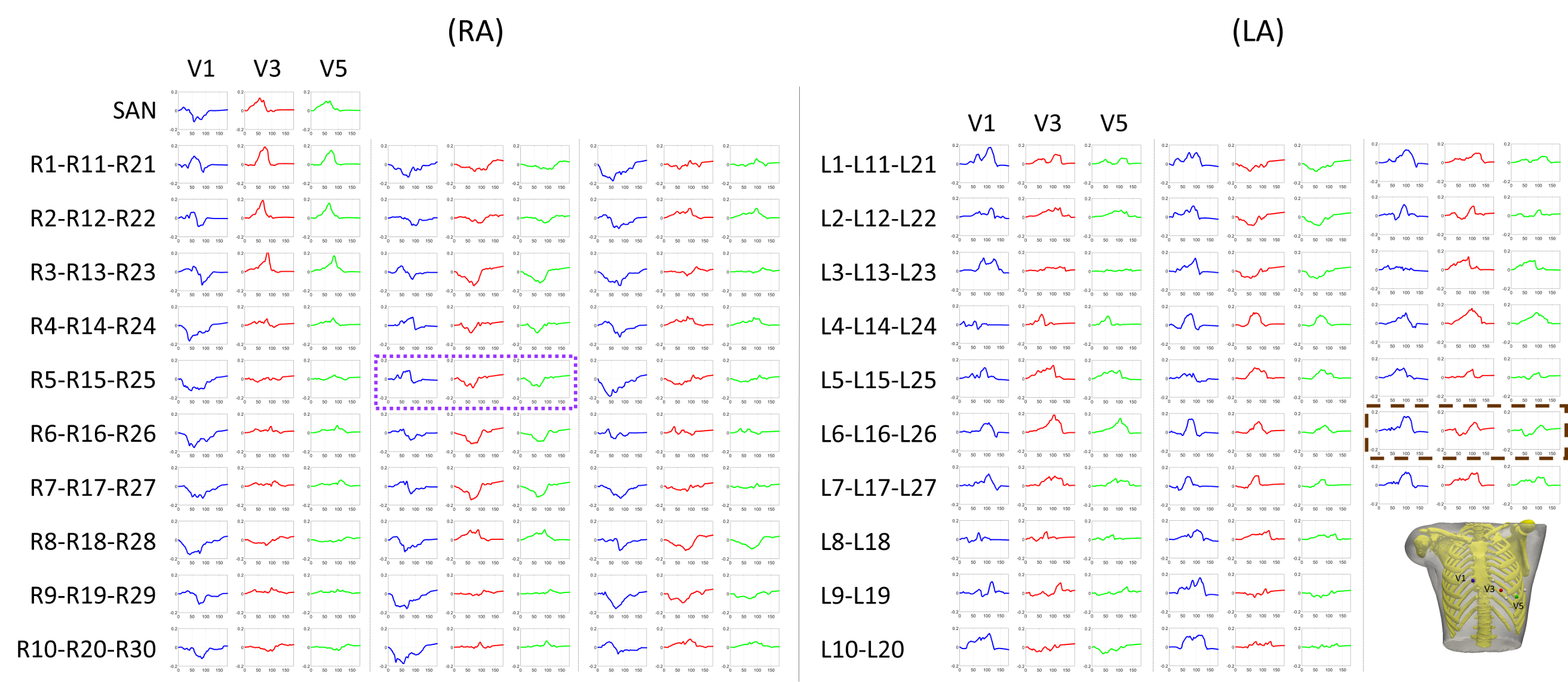

Supplement: S2 Fig — Position of the precordial leads on the torso surface and P-wave morphology registered at V1 (blue), V3 (red) and V5 (green) for the sites stimulated on RA (30 sites plus SAN) and the sites stimulated on LA (27 sites). The doted purple square, for example, identifies the P-waves registered at V1, V3 and V5 produced by the ectopic site R15. In the case of the dashed brown square, it identifies the P-waves produced by the ectopic site L26. (TIF) [file pone.0181263.s002.tif]

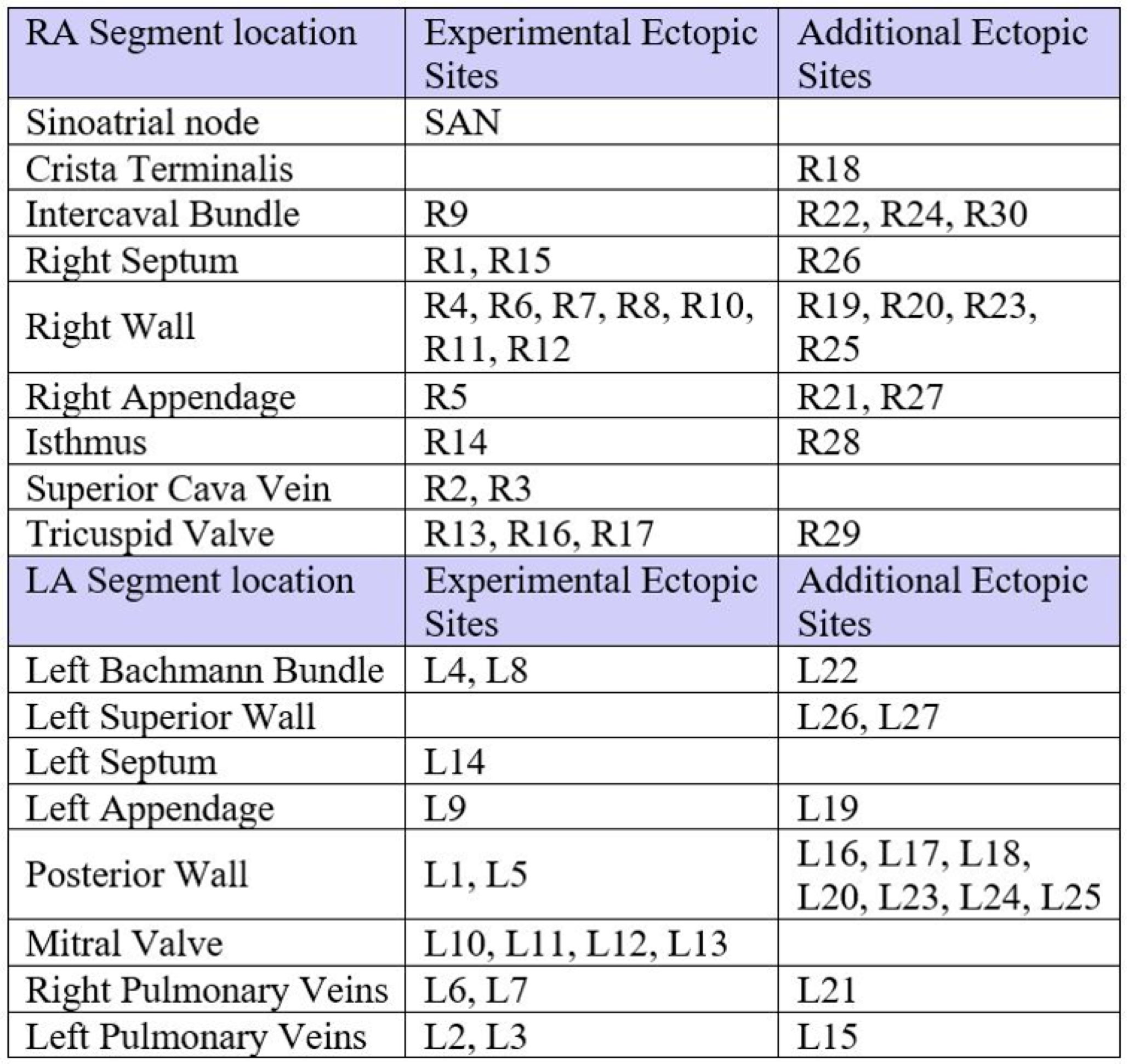

Supplement: S1 Table — 58 ectopic foci (including the SAN) grouped by RA and LA segment locations. The first 17 and 14 sites on RA and LA respectively (31 sites in total) were placed at the same positions used in previous experimental studies [8,10]. The additional 13 sites on each atrium (26 sites in total) were randomly selected to cover the whole atrial walls. (TIF) [file pone.0181263.s003.tif]
